# Do well-integrated species of an inquiline community have a lower brood predation tendency? A test using red wood ant myrmecophiles

**DOI:** 10.1186/s12862-016-0583-6

**Published:** 2016-01-19

**Authors:** Thomas Parmentier, Wouter Dekoninck, Tom Wenseleers

**Affiliations:** Laboratory of Socioecology and Socioevolution, KU Leuven, Naamsestraat 59, B-3000, Leuven, Belgium; Entomology Department, Royal Belgian Institute of Natural Sciences, Vautierstraat 29, B-1000, Brussels, Belgium

**Keywords:** Ant guests, Co-infection, *Formica rufa*, Niche partitioning, Parasite, Virulence, Co-evolution, Plastic defence

## Abstract

**Background:**

A host infected with multiple parasitic species provides a unique system to test evolutionary and ecological hypotheses. Different parasitic species associated with a single host are expected to occupy different niches. This niche specialization can evolve from intraguild competition among parasites. However, niche specialization can also be structured directly by the host when its defence strategy depends on the parasite’s potential impact. Then it can be expected that species with low or no tendency to prey on host brood will elicit less aggression than severe brood parasitic species and will be able to integrate better in the host system.

We examined this hypothesis in a large community of symbionts associated with European red wood ants (*Formica rufa* group) by testing the association between 1) level of symbiont integration (i.e. presence in dense brood chambers vs. less populated chambers without brood) 2) level of ant aggression towards the symbiont 3) brood predation tendency of the symbiont.

**Results:**

Symbionts differed vastly in integration level and we demonstrated for the first time that relatively unspecialized ant symbionts or myrmecophiles occur preferentially in brood chambers. Based on their integration level, we categorize the tested myrmecophiles into three categories: 1) species attracted to the dense brood chambers 2) species rarely or never present in the brood chambers 3) species randomly distributed throughout the nest. The associates varied greatly in brood predation tendency and in aggression elicited. However, we did not find a correlation for the whole myrmecophile community between a) brood predation tendency and host’s aggression b) integration level and host’s aggression c) integration level and brood predation tendency.

**Conclusions:**

Our results indicate that red wood ants did not act more hostile towards species that have a high tendency to prey on brood compared to species that are less likely or do not prey on brood. We show that potentially harmful parasites can penetrate into the deepest parts of a social insect fortress. We discuss these seemingly paradoxical findings in relation to models on coevolution and evolutionary arms races and list factors which can make the presence of potentially harmful parasites within the brood chambers evolutionary stable.

**Electronic supplementary material:**

The online version of this article (doi:10.1186/s12862-016-0583-6) contains supplementary material, which is available to authorized users.

## Background

Parasitism or the exploitation of one species by another species, is one of the most successful strategies in natural ecosystems [1]. The interactions between host and parasite often result in an evolutionary arms race where both partners develop adaptations and counter-adaptations against each other [2]. Most studies focus on the interaction between a single parasite and its host and address the adaptations and counter-adaptations. However, hosts are typically parasitized by an assemblage of species [3]. In such polyparasitism systems, the parasite's potential impact can vary substantially. Furthermore, parasites in such systems tend to specialize in different temporal and spatial niches associated with their host. For example, non-pollinating parasitic fig wasps present clear contrasts in oviposition timing, which promotes parasite co-existence [4] and trematodes avoid competition by parasitizing different parts of their snail host [5]. As an adequate defence response against parasites involves costs [6], it could be beneficial for the host if its level of aggression is hierarchically adjusted to the harmfulness of the symbiont. Such plastic defence has been demonstrated in studies with a small number of parasites associated with mammals, pine weevils and social insects [7–10], where hosts maximize the investment of costly defense strategies towards potential more harmful parasites, while potential less detrimental symbionts are tolerated.

A diverse group of organisms, ranging from commensals to true parasites, succeeded to penetrate into the well-defended nests of social insects [11, 12]. Those fortresses provide a unique environment with different microhabitats and abundant food resources. David Kistner categorized social insect symbionts in two major categories based on their behaviour: integrated species “which by their behaviour and their hosts’ behaviour can be seen as incorporated into their hosts' social life”; and non-integrated species, “which are not integrated into the social life of their hosts but which are adapted to the nest as an ecological niche [11].” Here we use the same nomenclature, but categorize symbionts rather on nest location than on their behaviour or host behaviour. In our definition, integrated species are able to penetrate into the dense brood chambers, whereas non-integrated species occur in sparsely populated nest chambers without brood at the periphery of the nest. There are indications that intraguild competition among social insect parasites can cause temporal niche specialization [13]. Alternatively, niche specialization can develop by a differential degree of tolerance of the host towards the symbionts. In that context, it is hypothesized that symbionts with lower potential costs are more integrated in the host’s colony and incite less aggression [14]. These predictions were supported in a study with the army ant *Leptogenys* [10]. Rove beetles preying on the host larvae elicited a strong aggression response. They were poorly integrated because they occur only at the edges of the colony. Rove beetles that do not prey on brood were better integrated in the colony. They did not receive aggression and were found in the central part of the nest. Some highly specialized myrmecophiles, however, do not follow these predictions. These species, such as larvae of the *Maculinea* butterflies, *Microdon* syrphid flies and *Lomechusa* rove beetles can integrate in the inner brood chambers of particular ant species without eliciting aggression [12, 15]. Those parasites have developed advanced chemical and behavioural adaptations to deceive their host [12, 16]. Those hosts and parasites are involved in a complex evolutionary arms race and their association may be stable due to frequency-dependent selection and geographic mosaic coevolution [17, 18]. However, in associations with less specialized species, which are the scope of this study, hosts could detect those intruders and adjust their aggression to the potential fitness costs that the parasite could incur on the host [10].

Our knowledge on life history strategies of social insect symbionts in species-rich host-macroparasite communities is weak and is mainly based on army ant host systems [11, 12, 19–21]. In parallel to the rich myrmecophile communities of tropical army ants [22], nests of European red wood ants (*F. rufa* group) are also hotspots for myrmecophile diversity [23]. However the organization of army ants and red wood ants (RWAs) is totally different. Army ants have an atypical life style: they do not construct permanent nests and regularly migrate to new temporal bivouacs. This organization also affects the symbionts as they have to coordinate their life cycle intimately with the host’s migrations [24, 25]. RWAs, on the other hand, construct a permanent, central nest. The aboveground part of their nest is a heap of organic thatch material, which provides plenty of hiding places for associated species and parasites throughout the mound. Because of these differences in the organization of their host, it is particularly interesting to compare the myrmecophile communities of army ants with those of nest-inhabiting red wood ants.

In this study, our ultimate aim was to test whether RWA myrmecophiles with a lower or no tendency to prey on brood are better integrated in the host ant colony. We tested the adaptive defence response of the host with a very large number of symbionts. We first determined three parameters for the different symbionts: (1) their level of integration in the colony (2) the level of host aggression elicited (3) their tendency to prey on ant brood. Linking these parameters allowed us to test the following hypotheses:

- Species with a lower level of brood predation elicit less aggression
  Some studies showed that ants are able to detect potential more harmful enemies and adjust their level of aggression concordantly [10, 26]. They argue that this hierarchy of aggression responses might promote colony fitness.
- Well-integrated species that reside in the dense brood chambers elicit lower level of aggression
  Better integrated symbionts are expected to elicit less aggression and are therefore able to stay in the dense brood chambers.
- Well-integrated species that live among the brood have a lower or no tendency to prey on brood
  From the perspective of the host, it is beneficial that it only tolerates species with low or no tendency to prey on brood, while severe brood parasites are only tolerated at the periphery of the nest or colony.

Consequently, species with low or no tendency to exhibit brood predation are tolerated and can integrate well into the colony, while species with a high brood parasite tendency are deterred to the edges of the colony by an elevated aggression response of the host.

## Methods

### Study system

A strikingly large number of obligate myrmecophiles can be found with the mound building European red wood ants (*Formica rufa* group) [23]. This myrmecophile community completely consists of rather unspecialized symbionts, except for the specialized, but rare myrmecophile *Lomechusa pubicollis* [27]. Specialized myrmecophiles (symphiles or true guests sensu Erich Wasmann [28]) are treated by the ants as members (fed and groomed) of the colony as a result of special glands (e.g. appeasement gland) and morphological (e.g. modified antennae) and behavioural adaptations (e.g. food soliciting). Unspecialized myrmecophiles (synechthrans and synoeketes sensu Erich Wasmann [28]), however, often look very similar to non-myrmecophile relatives and are ignored or treated with hostility [12, 27, 28]. Apart from obligate myrmecophiles, red wood ant mounds also host many facultative or occasional myrmecophiles. These arthropods mostly live away from ants, but can often be found in red wood ant mounds as well [23]. Red wood ant nests are heterogenic in worker distribution, with the largest abundances found in the inner brood chambers [29]. One could expect that more detrimental species would be recognized by the red wood ant hosts and are only tolerated at the outer edges of the nest away from the brood. However, it is not clear in what way other factors (e.g. abundance of hiding places, behavioural and chemical adaptations of symbionts) could affect this relation. To test our hypothesis for the red wood ant myrmecophiles community, we quantified three parameters: 1) level of integration 2) level of host ant aggression and 3) brood predation tendency, and examined whether they were linked. Hypothesis testing was done by using eight staphylinid beetle species (*Quedius brevis*, *Dinarda maerkelii*, *Thiasophila angulata*, *Notothecta flavipes*, *Lyprocorrhe anceps, Amidobia talpa*, *Leptacinus formicetorum*, *Stenus aterrimus*), two spiders (*Thyreosthenius biovatus*, *Mastigusa arietina*), one isopod (*Platyarthrus hoffmannseggi*), one springtail (*Cyphoderus albinus*), and five non-staphylinid beetle species: *Clytra quadripunctata* (Coleoptera: Chrysomelidae), *Monotoma angusticollis* (Coleoptera: Monotomidae), *Monotoma conicicollis* (Coleoptera: Monotomidae), *Dendrophilus pygmaeus* (Coleoptera: Histeridae) *Myrmetes paykulli* (Coleoptera: Histeridae). In addition, we collected *Porcellio scaber* in the mounds, which lives facultatively associated with red wood ants. All tested myrmecophiles are relatively unspecialized following the definition given above (Table 1). Myrmecophiles were caught by spreading nest material onto a large white tray in the field. We used the adult stage for all species, except for *C. quadripunctata* where we tested the larvae. Those larvae live in the nest and have a case in which they can hide. The adults of this species live on plants around the nests. After collecting myrmecophiles in the field, ants and their brood were gently placed back in the nest. Myrmecophiles were collected in seven red wood ant populations (description see [30]) across Western Flanders, Belgium and in one population in Boeschepe, France. Red wood ant populations consisted of *Formica rufa* and/or *Formica polyctena* mounds. Those closely related species have a very analogous colonial organization in the study area. Their myrmecophile community is likewise analogous [30].

**Table 1:** Proportion of individuals in brood chamber for the tested myrmecophiles

Species	Taxon	Myrmecophily	Host specifity	*N*	Proportion in brood chamber	95 % CI	*P*_corr_	Brood chamber
*Clytra quadripunctata*	Coleoptera (Chrysomelidae)	obligate	specialist	44	0.45	0.30–0.61	<0.001	attraction
*Thiasophila angulata*	Coleoptera (Staphilinidae)	obligate	specialist	91	0.37	0.27–0.48	<0.001	attraction
*Monotoma conicicollis*	Coleoptera (Monotomidae)	obligate	strict specialist	55	0.33	0.21–0.47	0.011	attraction
*Notothecta flavipes*	Coleoptera (Staphilinidae)	obligate	specialist	43	0.28	0.15–0.44	0.133	random
*Lyprocorrhe anceps*	Coleoptera (Staphilinidae)	obligate	specialist	54	0.28	0.16–0.42	0.102	random
*Platyarthrus hoffmannseggi*	Isopoda (Platyarthridae)	obligate	generalist	68	0.25	0.15–0.37	0.138	random
*Monotoma angusticollis*	Coleoptera (Monotomidae)	obligate	strict specialist	47	0.23	0.12–0.38	0.357	random
*Thyreosthenius biovatus*	Araneae (Linyphiidae)	obligate	specialist	54	0.22	0.12–0.36	0.357	random
*Dinarda maerkelii*	Coleoptera (Staphilinidae)	obligate	specialist	44	0.16	0.07–0.30	1.000	random
*Cyphoderus albinus*	Collembola (Cyphoderidae)	obligate	generalist	70	0.13	0.06–0.23	0.553	random
*Leptacinus formicetorum*	Coleoptera (Staphilinidae)	obligate	specialist	52	0.12	0.04–0.23	0.516	random
*Myrmetes paykulli*	Coleoptera (Histeridae)	obligate	specialist	44	0.11	0.04–0.25	0.514	random
*Amidobia talpa*	Coleoptera (Staphilinidae)	obligate	specialist	106	0.11	0.06–0.19	0.260	random
*Stenus aterrimus*	Coleoptera (Staphilinidae)	obligate	strict specialist	50	0.10	0.03–0.22	0.357	random
*Porcellio scaber*	Isopoda (Porcellionidae)	facultative	facultative	59	0.03	0.00–0.12	0.011	repulsion
*Dendrophilus pygmaeus*	Coleoptera (Histeridae)	obligate	specialist	26	0.00	0.00–0.13	0.043	repulsion
*Quedius brevis*	Coleoptera (Staphilinidae)	obligate	moderate	35	0.00	0.00–0.10	0.011	repulsion
*Mastigusa arietina*	Araneae (Dictynidae)	obligate	moderate	15	NA			

Attraction to or repulsion from the brood chamber was tested with an exact binomial two-sided test (deviation from a random distribution of 1/6 was tested). Reported P-values (*P*_corr_ *)* were adjusted for multiple testing by the Benjamini-Hochberg method (false discovery rate). *N* = number of individuals tested, for *D. pygmaeus* three individuals were re-used in different replicates. For *M. arietina*, all individuals were killed during the experiment and therefore no testing was done. 95 % CI: 95 % confidence. Host specifity based on supplementary Table in [23] (strict specialist: only records with RWAs, specialist: some records with non RWAs, but RWAs are the main host, moderate: records with RWAs, but distribution in non-RWAs probably important as well, generalist: myrmecophiles have no preference for a particular ant species, but are always found in presence of ants). Graphical representation of brood chamber association is given in Fig. 2

### Experiments

The experiments were performed between December 2012 and June 2015.

#### Experiment I: Level of integration

**Fig. 1:**
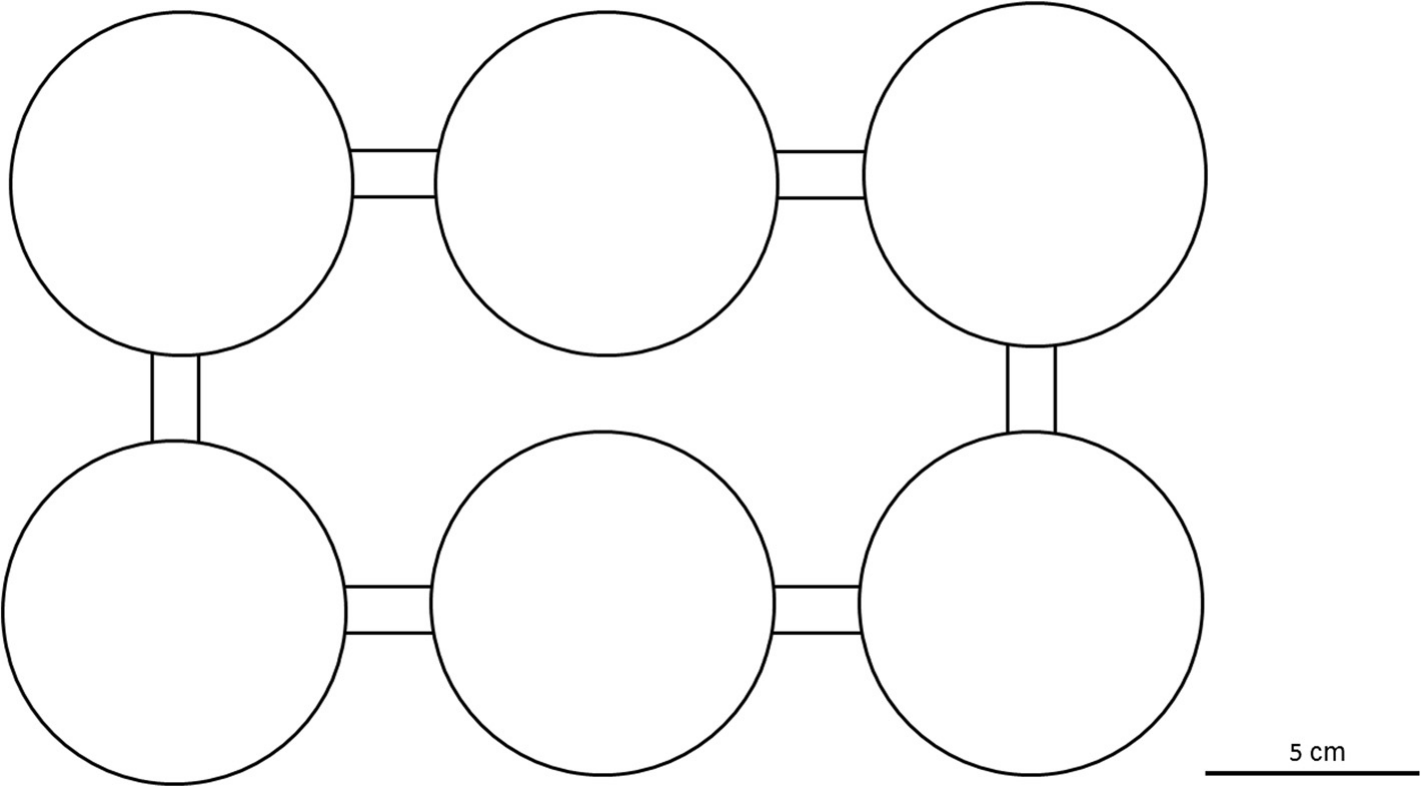
Schematic overview of the test nest. The nest consists of six chambers, in which each is connected with two other chambers

In this experiment, we wanted to test whether myrmecophiles occupied different niches in red wood ant nests. More specifically we were interested whether myrmecophiles preferred to stay in densely populated chambers with ant brood or in less densely populated areas without brood. Following our definition given above, integrated myrmecophiles penetrate into the densely populated chambers with brood, whereas poorly integrated species prefer sparsely populated chambers without brood. We constructed laboratory nests consisting of six round plastic pots (diameter 8 cm, height 5 cm) which were connected with plastic tubes (length 2 cm, inner diameter 1.1 cm). The pots and connections were arranged in such a way that every pot was connected with two other pots (Fig. 1). The bottom of the pots and connection tubes were filled with plaster of Paris (pots ca. 1 cm, tubes ca. 0.3 cm). We coated the inner walls of the pots with fluon to prevent ants and myrmecophiles from climbing up. In every pot (hereafter called chamber) we spread 10 g nest material (fine organic material) of a deserted *F. rufa* nest, to approach natural nest conditions and enabling myrmecophiles to hide. Transport and exchange of this nest material between the chambers was limited. All pots were sealed with a lid to prevent desiccation. We started each replicate by adding 360 *F. rufa* workers, 100 larvae of different sizes, 50 pupae and an egg pile (ca. 50 eggs/larvae) to the nest. Ants and their brood were collected in a supercolony in Boeschepe, France. After one day, myrmecophiles were apportioned randomly to the six chambers. The nest was placed in complete darkness to mimic natural conditions. Two days later, chamber openings were gently sealed with moist cotton plug and the nest was taken out of the darkness. The number of workers, brood and myrmecophiles were counted by spreading out the content of each chamber onto a large plastic tray with fluon coated walls. To distinguish *M. angusticollis* from *M. conicicollis*, we used a magnifier (4X, Eschenbach). Workers, brood and myrmecophiles that were found in the connection tubes were not considered. We replicated this experiment 16 times in total. We used different individuals for all myrmecophile species in each replicate, except for *D. pygmaeus*. For this species we found only three individuals and the same individuals were re-used in successive trials. The number of individuals per species recorded in each replicate at the beginning and at the end of the experiment is listed in Additional file 1: Table S1. Myrmecophiles for this experiment were collected in the Boeschepe population, but also in other red wood ant populations (*F. rufa* and *F. polyctena*) to increase our sample size. Aggression experiments for several myrmecophile species indicated that *F. rufa* workers did not act more aggressively towards myrmecophiles collected in *F. polyctena* colonies than towards myrmecophiles found in their own colony (Additional file 1: Table S3). Chemical analyses of the cuticular hydrocarbons confirm this lack of colony-specific and even RWA host-specific (i.e. individuals found in *F. rufa* do not differ from those found in *F. polyctena*) adaptation in all myrmecophiles tested in this paper (unpublished results). Therefore behaviour of myrmecophiles is expected not to be affected by the red wood ant colony of origin. Ant workers and brood were placed back in the host supercolony after the experiment.

#### Experiment II: Level of aggression elicited

We tested ant aggression toward myrmecophiles to study whether myrmecophiles elicited different aggression responses. Myrmecophiles and ants were collected in the same *F. rufa* supercolony in Westvleteren, except for *D. pygmaeus* and *M. arietina.* Those species were only found in *F. polyctena* populations. Based on the lack of RWA host-specific adaptation (unpublished results, Additional file 1: Table S3), we assume that these aggression tests of *D. pygmaeus* and *M. arietina* are comparable with those of the other myrmecophiles collected in the *F. rufa* colony (West-Vleteren). This was further confirmed with the high aggression of *F. polyctena* workers towards *M. arietina* found in the same colony, which was similar to the aggression of *F. rufa* towards those spiders (Additional file 1: Table S3). We used a small rectangular plastic arena (8 cm x 5.5 cm), filled with ca. 1 cm plaster of Paris and coated with fluon. Forty *F. rufa* workers were acclimatized for one hour to the arena. Then a myrmecophile was added and after ten seconds, the first twenty interactions (i.e. antennae of ant crossed the myrmecophile) with the ants were scored. Trials were performed in darkness under red light and were recorded with a video camera (SONY HDR-XR550VE). Videos were subsequently analysed in VirtualDub which allows to watch videos frame by frame. Ant aggression was scored by the proportion of aggressive interactions (acid spraying, chasing, biting, opening mandibles) out of the first 20 interactions. We used different myrmecophile individuals for each replicate, workers were re-used for several trials.

#### Experiment III: Brood predation tendency

Brood predation tendency of a myrmecophile species was quantified as the proportion of individuals that preyed on red wood ant eggs. We filled small plastic vials (diameter 4.5 cm) with ca. 1 cm of moistened plaster of Paris. Subsequently, we piled five red wood ant eggs in the centre and introduced a myrmecophile. Myrmecophiles were collected in different RWA populations in the study region described above. Eggs were collected in *F. rufa* colonies (Boeschepe and West-Vleteren). After one day, we checked whether the myrmecophile preyed on the eggs. For each myrmecophile species, we used different individuals in all replicates. We used acceptance of ant eggs (at least one egg eaten), rather than proportion of eggs eaten as the latter might be affected by the size of the myrmecophilic species. Individuals were starved for one day prior to the experiment. This index allowed us to classify myrmecophiles according to their tendency of brood predation. In the presence of ants, the success rate for the parasite might be lower. For the species that were attracted to the brood chamber in *Experiment I*, we also ran replicates with workers (five eggs and five workers in the same vial), to see if they still have a tendency to prey on ant brood.

### Data analysis

#### Experiment I: Level of integration

In all trials, ants stored the brood in one chamber (hereafter called the brood chamber). Chambers gradually spanned a large gradient in worker density with the brood chamber containing always the largest number of workers with an overall mean ± SD of 46.7 % ± 14.1 (see also Additional file 1: Table S2), reflecting the heterogeneity of worker density in natural wood ant nests ([31], personal observations).

Analyses were performed in R 3.2.1. Differences in association with the brood chambers in the myrmecophile community were compared using a generalized linear mixed model in a Bayesian setting with function blmer in R package ‘blme’ version 1.0.4 [32]. In contrast with generalized linear mixed models, this type of models can handle complete separation in a dataset by using a weak prior (http://ms.mcmaster.ca/~bolker/R/misc/foxchapter/bolker_chap.html). A part of our dataset was completely separated as some species were never observed in any of the brood chambers. The full model included the fixed factor ‘species’ and the random factor ‘replicate’. In addition, we incorporated an observation random factor to account for overdispersion [33]. A Type II Wald chisquare test was conducted with the Anova function in package ‘car’ version 2.1.0 [34] to assess whether species differed in level of integration (i.e. found in or outside the brood chamber). Post-hoc differences were tested by the glht function provided by package ‘multcomp’ version 1.4.1 [35]. We controlled the false discovery rate (multiple testing problem) by adjusting the *P*-values with the Benjamini-Hochberg method [36].

To test attraction or repulsion towards the brood chamber of a single species, we directly tested for each species whether the observed proportion of individuals in the brood chambers (pooled over the 16 replicates) deviated from a proportion of 1/6 with an exact binomial test. Indeed, in a six-chamber nest, we expect that a species with attraction to the brood chamber will have significant more occurrences than 1/6 in the brood chamber. In contrast, the occurrence probability in the brood chambers will be lower than 1/6 for species that avoid those chambers. We controlled the false discovery rate (multiple testing problem) of the multiple exact binomial tests by adjusting the *P*-values with the Benjamini-Hochberg method [36].

#### Experiment II and III: Level of aggression elicited and brood predation tendency

We ran a quasibinomial GLM with “species” as single explanatory factor and elicited aggression as dependent variable. Similarly, we tested with a quasibinomial GLM whether proportion of individuals preying on brood was significantly different. Significance was tested with a Likelihood Ratio chisquare test implemented in package *car*. Confidence intervals of aggression response and proportion individuals preying on eggs were calculated by the function confint (Table 2).

**Table 2:** Proportion aggressive interactions of ant workers towards myrmecophiles and proportion myrmecophile individuals preying on ant brood (= brood predation tendency) for different myrmecophile species

Species	Proportion aggressive interactions	*N*	95 % CI	Proportion individuals preyed on brood	*N*	95 % CI
*Amidobia talpa*	0.12	22	0.08–0.17	0.18	22	0.06–0.36
*Cyphoderus albinus*	0.00	15	0.00–0.02	0.00	15	0.00-NA
*Clytra quadripunctata*	0.01	10	0.00–0.03	0.67	24	0.48–0.83
*Dinarda maerkelii*	0.27	22	0.21–0.33	0.52	21	0.33–0.72
*Dendrophilus pygmaeus*	0.19	6	0.10–0.31	1.00	9	NA-1.00
*Lyprocorrhe anceps*	0.25	21	0.19–0.31	0.51	35	0.36–0.67
*Leptacinus formicetorum*	0.42	11	0.32–0.51	0.81	16	0.59–0.95
*Monotoma angusticollis*	0.03	20	0.01–0.06	0.68	25	0.49–0.83
*Mastigusa arietina*	0.73	12	0.64–0.81	0.10	10	0.01–0.36
*Monotoma conicicollis*	0.05	20	0.02–0.08	0.50	18	0.29–0.71
*Myrmetes paykulli*	0.23	18	0.13–0.25	0.67	21	0.46–0.83
*Notothecta flavipes*	0.63	21	0.56–0.70	0.96	23	0.83–1.00
*Platyarthrus hoffmannseggi*	0.05	20	0.03–0.09	0.60	20	0.39–0.79
*Porcellio scaber*	0.07	10	0.03–0.13	NA	NA.	NA
*Quedius brevis*	0.82	12	0.74–0.88	0.93	14	0.73–0.99
*Stenus aterrimus*	0.13	20	0.08–0.18	0.00	22	0.00-NA
*Thiasophila angulata*	0.45	35	0.40–0.50	0.98	41	0.90–1.00
*Thyreosthenius biovatus*	0.24	26	0.19–0.29	0.38	21	0.20–0.58

*N* number of individuals tested, *95 % CI* 95 % confidence interval, *NA* not available

#### Do well-integrated species of an inquiline community have a lower brood predation tendency?

We subdivided our main hypothesis in three parts: a) Do species with a lower tendency of brood predation elicit lower level of aggression? b) Do species that reside in the dense brood chambers elicit lower level of aggression? c) Do species that live among the brood have a lower tendency of brood predation? The three subhypotheses were tested by running both a Pearson product-moment and Spearman Rank correlation between a) brood predation tendency and level of aggression elicited b) level of integration and level of aggression elicited c) level of integration and brood predation tendency. We did not possess data on brood predation for *P. scaber* nor data on level of integration for *M. arietina* (all individuals were killed before the end of the experiment). Therefore, correlation between brood predation tendency and aggression elicited was run without *P. scaber* (*N*_species_ *=* 17), correlation between level of integration and aggression elicited was run without *M. arietina* (*N*_species_ *=* 17) and *c*orrelation between level of integration and brood predation tendency was done wihthout *M. arietina* and *P. scaber* (*N*_species_ *=* 16). In addition, we calculated the same correlations, but only focusing on the eight rove beetles (Staphylinidae) instead of all myrmecophiles.

Analyses were performed in R 3.2.1.

## Results

### Level of integration

**Fig. 2:**
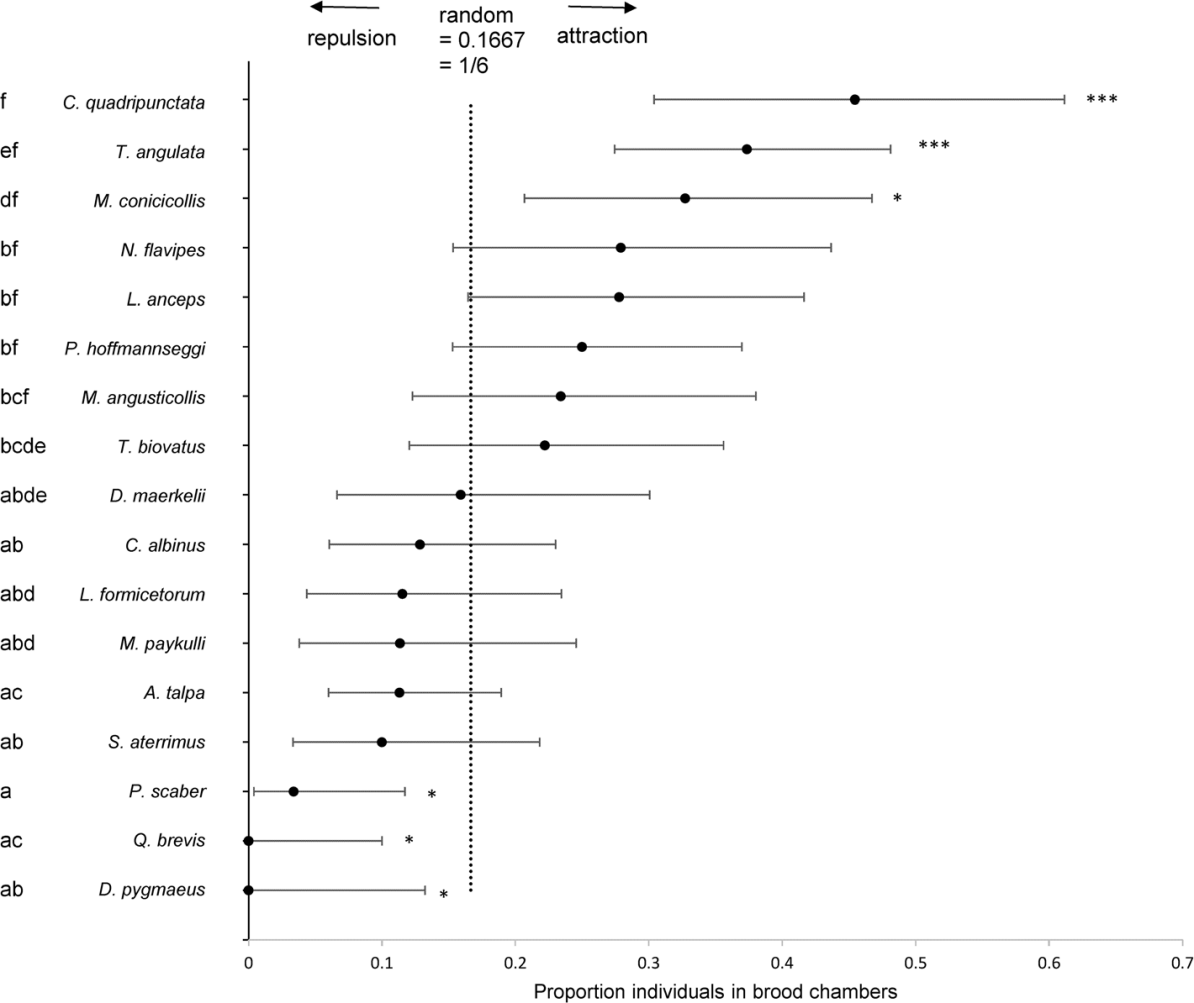
Level of integration of myrmecophiles. Proportion of individuals for different myrmecophilic species that were found in the brood chamber in the 6-chamber nest are given. Species attracted to the brood chambers (well-integrated) have proportions significant greater than 1/6, species that avoided the brood chambers (poorly integrated) have proportions significant lower than 1/6. Species without neither attraction or repulsion, have a more random distribution and the proportions in the brood chamber are not significantly different from 1/6. The observed proportion for a given myrmecophilic species was tested with an exact binomial two-sided test. P-values were corrected for multiple testing by the Benjamini-Hochberg method (false discovery rate), *P < 0.05, ***P < 0.001. Species with no letters in common are significant different at the α = 0.05 level (Bayesian generalized linear mixed model followed by Benjamini-Hochberg Post Hoc Tests)

Myrmecophiles differed significantly in preference for red wood ant brood chambers (BGLME, Chisq = 112.76, DF = 17, *P* < 0.001). Results of Benjamini-Hochberg Post-hoc tests are given with a letter code in Fig. 2. Myrmecophiles could be classified into three categories based on their association with the brood chambers: 1) attraction to the dense brood chambers 2) avoidance of the brood chambers and 3) random distribution (Fig. 2, Table 1). *Clytra quadripunctata* (mean proportion in brood chamber = 0.45, 95 % CI: 0.30–0.61, *P <* 0.001)*, T. angulata* (mean proportion in brood chamber = 0.37, 95 % CI: 0.27–0.48, *P <* 0.001) and *M. conicicollis* (mean proportion in brood chamber = 0.33, 95 % CI: 0.21–0.47, *P =* 0.011) were significantly attracted to the brood chambers (proportions in brood chamber significantly more than random 1/6 = 0.167 distribution). The highest attraction was found in the case-larvae of *C. quadripunctata*. The high attraction of this species to the dense brood parts of the nest was also directly observed in the field (sometimes they were also observed crawling on the mound). In the deep, central part of the nest, we also regularly found empty pupal cases which suggests that pupation also takes place in the heart of the nest. In contrast *Q. brevis* (mean proportion in brood chamber = 0.00, 95 % CI: 0.00–0.13, *P* = 0.043), *D. pygmaeus* (mean proportion in brood chamber = 0.00, 95 % CI: 0.00–0.10, *P* = 0.011) and the facultative associate *P. scaber* (mean proportion in brood chamber = 0.03, 95 % CI: 0.00–0.12, *P* = 0.011) significantly avoided the dense brood chambers (proportions in brood chambers significantly lower than random 1/6 = 0.167 distribution) (Table 1). *Q. brevis* and *D. pygmaeus* were even never observed in the brood chambers (Table 1). The spider *M. arietina* was always (15 individuals) killed before the end of the experiment, which might indicate that this species is not able to survive in a high density of workers without much hiding places. Field observations supported this apparent weak integration of the spider. It was never found in material with brood, but it was mainly found under pieces of bark in the nest. When disturbed, they ran rapidly away and hided in crevices and holes in the bark. Many distinct egg packets of this species (cf. [27]) could be found on the bark. Finally a group of myrmecophiles was rather randomly distributed in the nest, i.e. they were neither significantly attracted nor repelled from the brood chambers (Table 1).

### Level of aggression elicited and brood predation tendency

Ant aggression ranged vastly depending on the myrmecophile species (quasibinomial GLM, LR Chisq = 1563.5, *P* < 0.001) (Table 2). Some species such as *C. albinus, M. angusticollis* and *C. quadripunctata* were not or only very rarely attacked, while others such as *Q. brevis* and *M. arietina* were heavily attacked. The proportion of individuals that preyed on ant eggs varied greatly among myrmecophile species (quasibinomial GLM, LR Chisq = 199.72, *P* < 0.001) (Table 2). *Cyphoderus albinus* and *S. aterrimus* never preyed on ant eggs. In contrast, more than 90 % of the individuals of *N. flavipes*, *D. maerkelii*, *T. angulata*, *Q. brevis* and *D. pygmaeus* preyed on the ant eggs (Table 2). In the presence of ants, a similar (*C. quadripunctata N =* 9*,* proportion individuals preying on eggs = 0.67; *M. conicicollis, N =* 8, proportion individuals preying on eggs = 0.50) or lower proportion of egg predation (*T. angulata, N* = 10, proportion individuals preying on eggs = 0.70) was recorded for the three species that were attracted to the brood chambers compared with the tests without ants.

### Do well-integrated species of an inquiline community have a lower brood predation tendency?

**Fig. 3:**
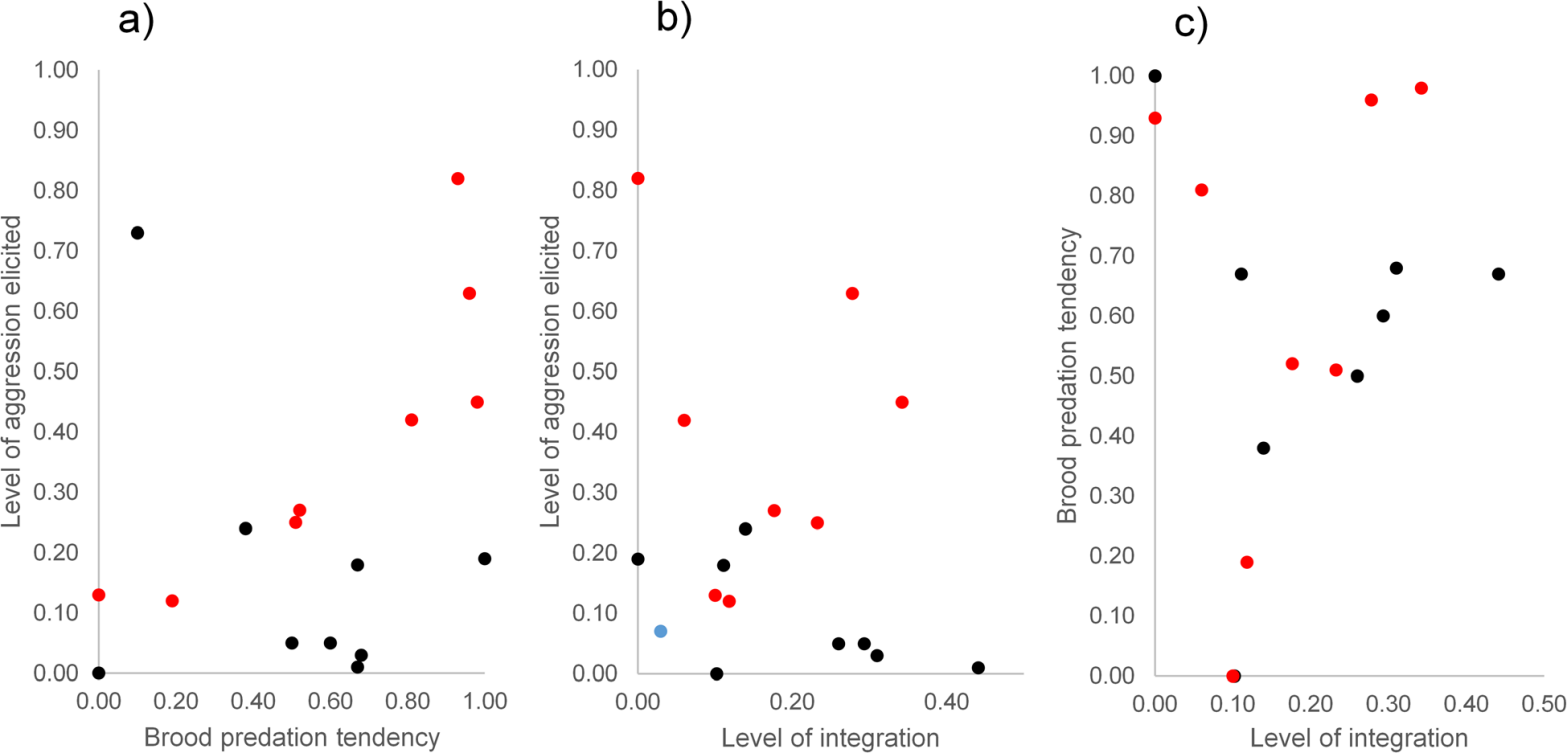
Relationship between brood predation tendency – level of elicited aggression - level of integration. **a** Relationship between level of elicited aggression and brood predation tendency (**b**) relationship between level of integration and level of elicited aggression and (**c**) relationship between level of integration and brood predation tendency. Level of aggression is the mean proportion of aggressive interactions out of 20 interactions with *F. rufa* workers (Exp.2). Brood predation tendency is the proportion of individuals that preyed on *F. rufa* eggs (Exp.3). Level of integration is the proportion of individuals integrated in the densely populated brood chamber (Exp. 1). Red points refer to staphylinid myrmecophiles, black points to non-staphylinid myrmecophiles, the blue point to the facultative myrmecophile *P. scaber*

Ants did not respond more aggressively towards myrmecophiles that have a higher brood predation tendency (Spearman’s rank correlation: *r* *=* 0.36, *P =* 0.153, Pearson’s product-moment correlation: *r* = 0.32, *P* = 0.206) (Fig. 3a). For example the severe brood parasite *C. quadripunctata* elicited hardly any aggression, whereas the low virulent spider *M. arietina* provoked a strong aggression response (Table 2). We did not find a correlation between the level of integration of the myrmecophiles and the aggression response of the ants (Spearman’s rank correlation: *r* = −0.22, *P =* 0.399, Pearson’s product-moment correlation: *r* = −0.22 *P =* 0.404), Those factors were also not linked, when we excluded the observation of the only facultative myrmecophile *P. scaber* (Spearman’s rank correlation: *r* = −0.22, *P =* 0.422, Pearson’s product-moment correlation: *r* = −0.25 *P =* 0.341) ((Fig. 3b). Illustrative for this lack of association is the high level of ant aggression towards some species (e.g. *T. angulata*) with a preference for the brood chambers. Finally, nest location preference was also not associated with the brood predation tendency of the myrmecophiles (Spearman’s rank correlation: *r* = 0.08, *P =* 0.761, Pearson’s product-moment correlation: *r* = 0.13, *P =* 0.624) (Fig. 3c). Here, some species with a high brood predation tendency (*C. quadripunctata, T. angulata*) preferred the dense brood chambers, whereas other species ranging from no to high brood predation tendency preferentially occurred away from the brood chambers or had no nest location preference.

When we only focused on the eight rove beetles, we found a strong positive correlation between worker aggression and brood predation tendency (Spearman’s rank correlation: *r* *=* 0.88, *P =* 0.007, Pearson’s product-moment correlation: *r* = 0.86, *P* = 0.007) (Fig. 3a red points). However, level of integration of rove beetles was not correlated with aggression response (Spearman’s rank correlation: *r* = 0.02, *P =* 0.977, Pearson’s product-moment correlation: *r* = − 0.09 , *P* = 0.831) and not with brood predation tendency (Spearman’s rank correlation: *r* *=* 0.38, *P =* 0.360, Pearson’s product-moment correlation: *r* = 0.27 *P* = 0.513). This means that ants responded more aggressively to rove beetles that are potentially more harmful, but they were not able to deter some harmful species (e.g. *N. flavipes* and *T. angulata*) from the brood chambers. In addition both rove beetles (*Q. brevis* and *L. formicetorum*) with a high (e.g. *Q. brevis*) and a low tendency (*S. aterrimus*) of brood predation had a relatively low integration.

## Discussion

In several multi-symbiont systems, it has been reported that symbionts are not homogenously distributed within the host system but occupy different spatial and temporal niches [10, 13, 37]. This is further supported by our data on red wood ant symbionts. We showed that those symbiont species are indeed heterogeneously distributed across their host nests. More specifically, some species were attracted to the densely populated brood chambers, whereas rather poorly integrated species clearly avoided those dense brood chambers. Another group did not appear to be attracted or repulsed by the dense brood chambers. We showed here for the first time the attraction of relatively unspecialized (synechthrans and synoeketes sensu Wasmann [28]) species towards the brood chambers in social insects. Generally it is assumed that only specialized (symphiles sensu Wasmann [28]) species are able to settle among the brood in ant colonies [12].

Niche selection in multiple symbiont systems can result from avoiding competition with other symbionts (described as niche partitioning) [4, 13]. However, in several host-multiparasite systems, it has been reported that the host adjusts its defence to the potential negative impact of the symbiont [7–10]. Niche selection of symbionts can then be an outcome of differential host-symbiont interaction rather than resulting from competition among symbionts. In this case, niche occupation or level of integration results from a varying tolerance of the host for different symbionts. For example, the army ant *Leptogenys* behaves more aggressively towards some associated rove beetles than to others. Therefore the less aggressed species can thrive in the centre of the colony, whereas the other species are only tolerated at the margins of the colony. From an evolutionary point of view, it is a good strategy to be more aggressive to symbionts with a high brood predation tendency and chase them away from the brood chambers. This was hypothesized in [14] and supported in [10]. In our experiments, ants did act more aggressively towards rove beetles with a higher potential for brood predation and more peaceful to species with no or low brood predation tendency. However, this association was absent, when we look at the entire myrmecophile community, including non-staphylinid myrmecophiles. For example, the spider *M. arietina* had a very low tendency for brood predation, but was heavily persecuted in the aggression experiments and bitten to death in all nest location preference trials. Moreover, our results did not show a correlation between nest location and brood predation tendency for staphilinids and the myrmecophile community as a whole. Species with a preference for the brood chambers were even characterized by a relatively high brood predation tendency. They are not only potentially dangerous, but incur real costs, as the presence of ant workers did not stop them from parasitizing on the brood. Species that avoided brood chambers ranged from non-brood predators to species with a high brood predation tendency. There was also no correlation between nest location and ant’s aggression response for staphylinids and the myrmecophile community as a whole. In contrary to the expectations that species in the brood chambers will provoke less aggression, we found that some species that hardly elicited an aggressive response stayed away from the inner brood chambers or had a more random distribution. Some species (e.g. *T. angulata*), on the other hand, elicited a strong response, but still preferred the dense brood chambers and managed to cope with this highly stressful conditions.

It is puzzling how symbionts with a high brood predation tendency succeed to live within the dense brood chambers without being repulsed. At the proximate level, the tested myrmecophiles employ different strategies to overcome ant defence. In contrast with army ants, wood ant mound architecture provide a plethora of hiding places. Small and slender myrmecophiles, especially rove beetles can quickly squeeze in small holes and cracks when aggressed. Severe brood parasitic rove beetles could therefore, in spite of being recognized as potential harmful, integrate well in the colonies. *Clytra quadripunctata*, the myrmecophile with the highest preference for the brood chamber, on the contrary, relies on a morphological adaptation. When attacked, they withdraw in their protective case and seal the opening with their well armoured head [27]. *Monotoma* beetles are slow-moving small beetles and retract their legs when attacked which render them difficult to detect. Future research will also reveal whether chemical strategies such as chemical insignificance are involved in the integration of brood predators [38–40].

At the ultimate level, the lack of rejection of brood predators in the brood chambers can be explained by two theoretical models that are not mutually exclusive. “The evolutionary lag hypothesis” states that no genetic variation in defence strategies emerged in the host at this point. But once available, efficient defence will spread and become fixed. This hypothesis assumes that parasite repulsion is beneficial from the host’s perspective. Here the parasite is currently the winning partner in an ongoing evolutionary arms race and it only takes time before the host evolves counter-adaptations [41, 42]. However, when a host is infected by multiple parasites, as in our ant-myrmecophile study system, defence strategies can be a compromise to different parasites and clear co-evolutionary traits are consequently harder to identify [42]. Alternatively, the evolutionary equilibrium hypothesis predicts that owing to the costs involved with parasite repellence, parasite acceptance or tolerance counter-intuitively can become beneficial. The arms-race comes here to a standstill in a stable equilibrium and the observed defence strategy is than determined by a balance of parasite load and the costs to defend against those parasites [43–45]. For example, the Jacobin cuckoo (*Clamator jacobinus*) lay a non-mimetic egg in the nest of its host. The host cannot eject or puncture the egg because it is too large (double size of host egg) and has a thick shell. The host can still avoid brood parasitism by abandoning the nest, but this entails high costs due to an elevated predation and parasitism risk later in the season which exceed the costs for accepting the cuckoo egg. Therefore a non-mimetic cuckoo egg and the lack of a host defence response will here be a stable equilibrium [46]. Defence against parasitic myrmecophiles could also be costly for ants. First, regular task switching to defensive roles involve costs for workers due to time needed to perform defensive behaviour and energy costs owing to shifts in behavioural state [47, 48]. Second, myrmecophiles and especially rove beetles may emit repellent, toxic, or alarm inducing chemicals when aggressed [49, 50] and might interfere as such normal colony routine and organization.

The presence of brood predators among the brood can dramatically affect colony fitness [51, 52]. However, different mechanisms can lower the cost of the parasites on their red wood ant host. First, wood ant nests provide a multitude of food resources. We demonstrated that most myrmecophiles only facultatively feed on ant brood [53]. Second, red wood ant parasites control each other by intraguild predation [53]. Brood predation can also be lower for some species in presence of ants implying that ants partly deter some brood predators [54]. Finally, red wood ants nests regularly abandon their nest and construct new mounds on another location. However untested yet, it is argued that nest displacement can be an effective tool to control parasite infection [10].

## Conclusions

This study provides a unique insight in the different strategies of social insect symbionts and the interactions with their host. We demonstrated that symbionts associated with ants differ greatly in the level of integration in the host nest. We showed that unspecialized species can thrive in the densely populated brood chambers, whereas others are poorly integrated and prefer scarcely populated chambers. Moreover we demonstrated that myrmecophiles have a varying degree of brood predation tendency. Remarkably, a myrmecophile’s level of integration in the colony or its brood predation tendency is not linked with the intensity of the aggression response of the host. We found that some potential brood predators are poorly integrated, but others manage to live and are attracted to the brood chambers. Some brood predators appear thus to be in the lead in an evolutionary arms race with their host, as the host does not recognize them as a dreadful foe or do not manage to repel them from the brood chambers. Further investigations will lead to a better understanding in the dynamics between host and parasite and will explore mechanisms which make the presence of brood predators among the brood evolutionary stable.

## Additional file

Additional file 1: Table S1. Number of individuals recorded at the end of the experiment in the different replicates is given per myrmecophile species. **Table S2.** Distribution of workers in the test nest chambers over the different replicates. Brood chambers always supported the largest number of workers and are marked in yellow. **Table S3.** Proportion aggressive interactions of F. rufa workers (West-Vleteren) towards myrmecophiles found in F. polyctena colonies (“polyctena” treatment) compared with aggression of F. rufa workers (West-Vleteren) towards myrmecophiles found in the same F. rufa colony. (PDF 97 kb)
