# Supplementary material for: Do well-integrated species of an inquiline community have a lower brood predation tendency? A test using red wood ant myrmecophiles
Source: BMC Evol Biol. 2016 Jan 19;16:12. doi: 10.1186/s12862-016-0583-6 (PMC4717668; doi:10.1186/s12862-016-0583-6)
Supplement: Additional file 1: Table S1. — Number of individuals recorded at the end of the experiment in the different replicates is given per myrmecophile species. Table S2. Distribution of workers in the test nest chambers over the different replicates. Brood chambers always supported the largest number of workers and are marked in yellow. Table S3. Proportion aggressive interactions of F. rufa workers (West-Vleteren) towards myrmecophiles found in F. polyctena colonies (“polyctena” treatment) compared with aggression of F. rufa workers (West-Vleteren) towards myrmecophiles found in the same F. rufa colony. (PDF 97 kb) [file 12862_2016_583_MOESM1_ESM.pdf]

Additional file 1

Table S1. Number of individuals recorded at the end of the experiment in the different replicates is given per myrmecophile species. Number of individuals at the beginning of the experiment is given in brackets below. All individuals of *M. arietina* were killed before the end of the experiment. Predation of other myrmecophiles on *C. albinus* explains its low survival [54].

	A. talpa	*C.quadripunctata*	*C. albinus*	*D.pygmaeus*	*D.maerkelii*	*L. formicetorum*	*L. anceps*	*M. angusticollis*	*M. conicicollis*	*M.paykulli*	*M. arietina*	*N. flavipes*	*P.hoffmannseggi*	*P. scaber*	*Q. brevis*	*S. aterrimus*	*T. angulata*	*T. biovatus*
Replicate 1	10 (11)		5 (20)	1 (1)	2 (2)	4 (4)	4 (4)	7 (7)	4 (4)	4 (4)	0 (5)	5 (5)	2 (5)	5 (5)	3 (3)	3 (3)	8 (8)	3 (3)
Replicate 2	6 (7)		7 (20)	1 (1)	1 (1)	2 (2)	2 (3)	4 (4)	4 (4)	4 (4)	0 (3)	4 (5)	5 (5)	4 (5)	3 (3)	2 (3)	6 (6)	2 (4)
Replicate 3	9 (9)		8 (20)	1 (1)	1 (1)	2 (2)	4 (4)	3 (4)	3 (3)	3 (3)	0 (4)	4 (5)	5 (5)	4 (5)	3 (3)	3 (3)	5 (5)	4 (4)
Replicate 4	13 (13)		9 (20)	1 (1)	2 (3)		2 (3)	4 (5)	4 (5)	3 (4)	0 (2)	3 (3)	2 (5)	4 (5)	3 (3)	4 (4)	3 (5)	6 (6)
Replicate 5	10 (11)		6 (20)	1 (1)	2 (2)	8 (8)	4 (4)	3 (5)	3 (5)	2 (4)		2 (3)	6 (6)	5 (5)	3 (3)	3 (3)	2 (5)	3 (6)
Replicate 6	8 (10)		3 (20)	1 (1)	2 (2)	1 (1)	3 (3)	2 (4)	6 (6)	3 (4)		3 (3)	3 (5)	3 (5)	3 (3)	2 (3)	3 (5)	6 (6)
Replicate 7	3 (10)		5 (20)	3 (3)	3 (3)	5 (6)	6 (6)	2 (3)	5 (5)	6 (4)		4 (3)	4 (4)	4 (5)	2 (3)	4 (4)	10 (10)	4 (5)
Replicate 8	9 (9)	6 (6)	3 (20)	1 (1)	5 (5)	2 (2)	3 (4)	2 (3)	3 (3)	3 (3)		3 (3)	5 (5)	3 (5)	3 (3)	3 (3)	7 (7)	2 (3)
Replicate 9	9 (10)	7 (7)	3 (19)	1 (1)	6 (6)	1 (2)	3 (3)	4 (5)	2 (2)	3 (3)		4 (4)	5 (5)	2 (4)	3 (3)	3 (3)	5 (5)	3 (3)
Replicate 10	9 (10)	7 (7)	0 (15)	3 (3)	4 (5)	5 (5)	3 (3)	2 (2)	2 (2)	2 (2)			4 (5)	4 (4)	3 (3)	4 (4)	6 (6)	3 (3)
Replicate 11	1 (5)	5 (5)		1 (1)	2 (2)	1 (3)	4 (4)	2 (2)	2 (2)	1 (1)		1 (2)	5 (5)	4 (4)	0 (1)	4 (4)	6 (6)	2 (3)
Replicate 12	3 (5)	4 (5)		1 (1)	2 (2)	2 (3)	2 (2)	1 (1)	2 (2)	1 (1)		2 (2)	7 (7)	3 (4)	1 (1)	1 (3)	10 (10)	3 (4)
Replicate 13	3 (5)	5 (5)		1 (1)	2 (2)	0 (3)	3 (3)		2 (2)	1 (1)		1 (2)	5 (5)	5 (5)	1 (1)	4 (4)	5 (5)	2 (3)
Replicate 14	6 (6)	3 (5)	21 (30)	3 (3)	4 (5)	9 (11)	4 (5)		6 (6)	3 (5)		3 (5)	7 (7)	3 (5)	2 (2)	4 (5)	3 (5)	5 (6)
Replicate 15	4 (4)	2 (3)		3 (3)	3 (3)	4 (8)	3 (3)	2 (4)	2 (2)	2 (3)		2 (3)	3 (5)	3 (5)	1 (2)	3 (3)	7 (7)	4 (6)
Replicate 16	3 (7)	5 (5)		3 (3)	3 (3)	6 (7)	4 (5)	9 (9)	5 (6)	3 (3)	0 (1)	2 (3)		3 (4)	1 (1)	3 (3)	5 (5)	2 (4)
Total *N* individuals	**106** (132)	**44** (48)	**70** (224)	**26** (26)	**44** (47)	**52** (67)	**54** (59)	**47** (56)	**55** (59)	**44** (49)	**0** (15)	**43** (51)	**68** (79)	**59** (75)	**35** (38)	**50** (55)	**91** (100)	**54** (69)

Table S2. Distribution of workers in the test nest chambers over the different replicates. Brood chambers always supported the largest number of workers and are marked in yellow. Total workers at the end of the experiment is function of the number of workers (=360) at the start of the experiment, dead workers and workers emerged from pupae during the experiment.

	Chamber 1	Chamber 2	Chamber 3	Chamber 4	Chamber 5	Chamber 6	Total Workers
Replicate 1	7	**252**	32	12	24	41	368
Replicate 2	13	**137**	125	17	7	38	337
Replicate 3	6	13	4	**249**	19	19	310
Replicate 4	110	**130**	28	15	9	8	300
Replicate 5	9	44	67	32	32	**136**	320
Replicate 6	39	26	36	94	**115**	24	334
Replicate 7	33	**141**	61	40	37	49	361
Replicate 8	73	**145**	40	41	26	51	376
Replicate 9	27	66	38	41	**105**	32	309
Replicate 10	20	20	**207**	16	23	33	319
Replicate 11	**154**	152	28	7	16	17	374
Replicate 12	33	12	19	74	74	**135**	347
Replicate 13	**137**	81	12	66	23	7	326
Replicate 14	**140**	37	31	70	18	23	319
Replicate 15	62	69	14	71	**161**	9	386
Replicate 16	88	18	32	59	42	**100**	339

Is the aggression response of *F. rufa* towards the tested myrmecophiles elevated when these myrmecophiles are collected in other red wood ant nests?

In this behavioural experiment, we tested whether the aggression response of *F. rufa* (West-Vleteren colony) workers towards myrmecophiles collected in the same West-Vleteren colony ("local rufa treatment") was different from the aggression response of those *F. rufa* workers (of the same West-Vleteren colony) towards myrmecophiles collected in *F. polyctena* colonies ("polyctena treatment"). Experiments followed the protocol outlined in the material and method section "Experiment II: Level of aggression elicited". For every replicate different myrmecophile individuals were introduced in the test arena. In total, we compared aggression in the "local rufa" treatment with the "polyctena" treatment for 12 out of 18 myrmecophiles that were tested in the main manuscript. Data on aggression in the local rufa treatment can also be found in Table 2. For every tested myrmecophile, we ran a quasibinomial GLM to test whether the proportion of aggressive interactions of F. *rufa* workers in the "local rufa treatment" differed from the "polyctena treatment". Significance was tested with a Likelihood Ratio chisquare test implemented in the R package car. P-values are adjusted for multiple testing (Benjamini and Hochberg, false discovery rate, [37]).

Aggression of *F. rufa* workers was similar in the "local rufa" and "polyctena treatment" for the 12 tested species (Table B.1). If there was local or RWA host-specific adaptation, you would expect that *F. rufa* workers would act more aggressively toward myrmecophiles found in *F. polyctena* mounds than toward inquilines found in their own colony. *M. paykulli* has the highest chemical similarity (unpublished results) with its host out of the 18 myrmecophiles tested in the main document. Nevertheless, these behaviour data also suggest that this species lack RWA host-specific chemical adaptation (Table B.1).

Table S3. Proportion aggressive interactions of *F. rufa* workers (West-Vleteren) towards myrmecophiles found in *F. polyctena* colonies ("polyctena" treatment) compared with aggression of *F. rufa* workers (West-Vleteren) towards myrmecophiles found in the same *F. rufa* colony. *N* = number of individuals tested, 95% CI: 95% confidence interval. *P* = uncorrected *P*-values, P_{cor} = *P*-values controlled with the Benjamini-Hochberg method (multiple testing problem). Note that the CI for myrmecophiles in the local treatment can be slightly different from those given in Table 2 in the main file. This is because the CI are estimated in different models. Here we used per species a quasibinomial model with treatment as factor, in Table 2 of the main file we used one quasibinomial model with species as factor.

Species	"*polyctena*" treatment			"Local *rufa*" treatment			*P*	P_{cor}
	Proportion aggressive interactions	*N*	95% CI	Proportion aggressive interactions	*N*	95% CI		
Amidobia talpa	0.08	21	0.05-0.12	0.12	22	0.08-0.16	0.141	0.524
Cyphoderus albinus	0.01	20	0.00-0.02	0.00	15	0.00-0.01	0.257	0.524
Lyprocorrhe anceps	0.28	14	0.21-0.35	0.25	21	0.19-0.30	0.470	0.564
Monotoma angusticollis	0.05	25	0.03-0.07	0.03	20	0.02-0.05	0.09	0.524
Mastigusa arietina	0.73	12	0.64-0.80	(*)				
Monotoma conicicollis	0.05	17	0.03-0.08	0.05	20	0.03-0.07	0.736	0.803
Myrmetes paykulli	0.14	16	0.10-0.20	0.18	18	0.13-0.24	0.302	0.524
Notothecta flavipes	0.52	24	0.45-0.59	0.63	21	0.56-0.71	0.035	0.420
Platyarthrus hoffmannseggi	0.04	20	0.02-0.07	0.05	20	0.03-0.09	0.452	0.564
Porcellio scaber	0.07	15	0.03-0.13	0.07	10	0.02-0.15	0.994	0.994
Quedius brevis	0.74	8	0.60-0.85	0.82	12	0.71-0.90	0.318	0.524
Thiasophila angulata	0.50	31	0.43-0.56	0.45	35	0.39-0.51	0.328	0.524
Thyreosthenius biovatus	0.28	26	0.22-0.34	0.24	26	0.18-0.29	0.349	0.524

(*) The high aggression response of *F. rufa* towards *M. arietina* (cf. Exp. I: all 15 individuals were killed before the end of the experiment, Exp II: proportion aggressive interactions = 0.73) was very striking. Here, there could also be an effect of host or colony specific chemical adaptation. Unfortunately this species was only found in *F. polyctena* colonies, so we were unable to test whether *F. rufa* provoked higher aggression towards this species when found in the same colony or originating from *F.* polyctena. But there was no chemical similarity at all with *F. polyctena*

(unpublished GCMS) for this species. Moreover we also tested aggression of *F. polyctena* workers following the protocol of Exp. II towards 2 *M. arietina* individuals found in the same *F. polyctena* colony. Aggression was likewise very high:

- *M. arietina* ind. 1: proportion aggressive interactions = 0.65: (of which 4 biting interactions)
- *M. arietina* ind. 2: proportion aggressive interactions = 0.80: (of which 6 biting interactions)

Individual 1 was even deadly wounded during the aggression tests and died shortly after. Given these data, we assume that *F. rufa* workers would behave in a similar way when *M. arietina* was found in the same colony.
